# Prediction of Magnetic Fields in Single-Phase Transformers Under Excitation Inrush Based on Machine Learning

**DOI:** 10.3390/s25134150

**Published:** 2025-07-03

**Authors:** Qingjun Peng, Hantao Du, Zezhong Zheng, Haowei Zhu, Yuhang Fang

**Affiliations:** 1 Electric Power Research Institute of Yunnan Power Grid Corporation, Kunming 650127, China; qingjunpeng@ieee.org; 2 School of Resources and Environment, University of Electronic Science and Technology of China, Chengdu 611731, China; 202322070215@std.uestc.edu.cn (H.D.); 202052070331@std.uestc.edu.cn (H.Z.); yuhangfang@uestc.edu.cn (Y.F.)

**Keywords:** prediction of magnetic fields, machine learning, finite element analysis, transformer

## Abstract

With the digital transformation of power systems, higher demands are being placed on smart grids for the timely and precise acquisition of the status of transmission and transformation equipment during operational and maintenance processes. When a transformer is energized under no-load conditions, an excitation inrush phenomenon occurs in the windings, posing a hazard to the stable operation of the power system. A machine learning approach is proposed in this paper for predicting the internal magnetic field of transformers under excitation inrush condition, enabling the monitoring of transformer operation status. Experimental results indicate that the mean absolute percentage error (MAPE) for predicting the transformer’s magnetic field using the deep neural network (DNN) model is 4.02%. The average time to obtain a single magnetic field data prediction is 0.41 s, which is 46.68 times faster than traditional finite element analysis (FEA) method, validating the effectiveness of machine learning for magnetic field prediction.

## 1. Introduction

With the digital transformation of power grid systems, there are increasing demands on acquiring and maintaining transformer states in digital twin grids. Timely and accurate state acquisition of transformers is crucial for ensuring power grid equipment safety and is a key challenge in enhancing power grid intelligence.

Analyzing the distribution characteristics of physical fields like electrical, magnetic, thermal, mechanical, and fluid within transformers, along with their interrelationships, couplings, and interactions, is vital for transformer fault diagnosis. Finite Element Analysis (FEA) is the primary method used for analyzing these physical fields in transformers [1,2]. However, FEA is difficult for meeting real-time performance requirements. To address the time-consuming and high computational resource demands of traditional FEA method, we introduce machine learning methods which can accelerate the solution of FEA equations [3]. Machine learning exhibits superior feature extraction capabilities, enabling it to handle complex mappings among numerous parameters. Additionally, it allows for the design of network models according to different types of faults. Zhang et al. developed a novel self-supervised graph feature enhancement and scale attention-driven node-level fault classification framework to enhance the diagnosis performance [4]. Also, their team introduced a multiscale channel attention-driven graph dynamic fusion network for mechanical fault diagnosis [5]. By utilizing machine learning’s feature extraction strengths, mapping the relationship between equation solution spaces and finite element modeling parameters can significantly reduce simulation time and costs.

Vurtur et al. mapped a simplified dataset from real-time structural measurements to a high-fidelity FEA model of the same system, achieving the prediction of stress distribution within the vehicle structure, which in turn enhanced the capability to develop specified vehicle safety and efficient maintenance plans [6]. Kohar et al. utilized a finite element model of dynamic axial extrusion of rectangular tubes in vehicle crashworthiness applications, altered the size and wall thickness of the finite element model, and employed a 3D Convolutional Neural Network (CNN) autoencoder and Long Short-Term Memory (LSTM) networks to predict the mesh deformation and force-displacement response. This method is computationally faster than the traditional finite element methods and exhibits good accuracy [7]. Ford et al. input random biphasic microstructures into an FEA program to determine the elastic modulus, Poisson’s ratio, and corresponding stresses. Prior to training and testing Artificial Neural Network (ANN) and Random Forest ensemble machine learning methods, the two-point correlation function and Principal Component Analysis (PCA) were applied to the microstructures. The experimental results indicate that machine learning methods can accurately predict the homogeneous elastic properties [8]. Chugh et al. effectively applied machine learning techniques to problems requiring optimization of a large number of parameters, and by combining finite element simulation with machine learning techniques, they predicted the mode effective index, power constraints, and coupling lengths of different integrated photonic devices. The optimized machine learning model can predict the output of different device parameters faster than direct numerical simulation techniques [9]. Babes et al. exploited a fuzzy neural network to approximate the unknown converter nonlinear dynamics due to changes in the input voltage and loads. All results confirm fast reference tracking capability with low overshoots and robustness against disturbances while comparing with the traditional fast terminal synergetic controller [10]. Larouci et al. used the seagull optimization algorithm, crow search algorithm, tunicate swarm algorithm, and firefly algorithm to tackle the dynamic combined economic environmental dispatch problems with variable real transmission losses [11].

Currently, although traditional finite element simulation methods have matured in research and are suitable for physical field simulations of most equipment in the current power industry, they struggle to balance computational efficiency while ensuring simulation accuracy, which fails to meet the demands of digital twin grid construction. This paper introduces machine learning into the field of transformer magnetic field simulation, providing strong theoretical support for rapid prediction of magnetic fields.

In this paper, we propose a method that combines various machine learning models with PCA to address the time-consuming and computationally intensive nature of FEA simulations. This method aims to rapidly predict internal magnetic fields of transformers during excitation inrush conditions. The predictive results of the machine learning model closely approximate the magnetic fields of the overall transformer and internal components in the actual power grid. First, the influence of input features was evaluated through a ranking of importance, thereby determining the input dimensionality of the model. The change of the internal electrical parameters of the transformer under excitation inrush conditions were analyzed, which elucidated their impact on the transformer’s magnetic field, thereby enhancing the interpretability of the model.

Subsequently, data preprocessing was conducted based on the magnetic field within the transformer, and the magnetic field prediction model was established.

**Figure 1:**
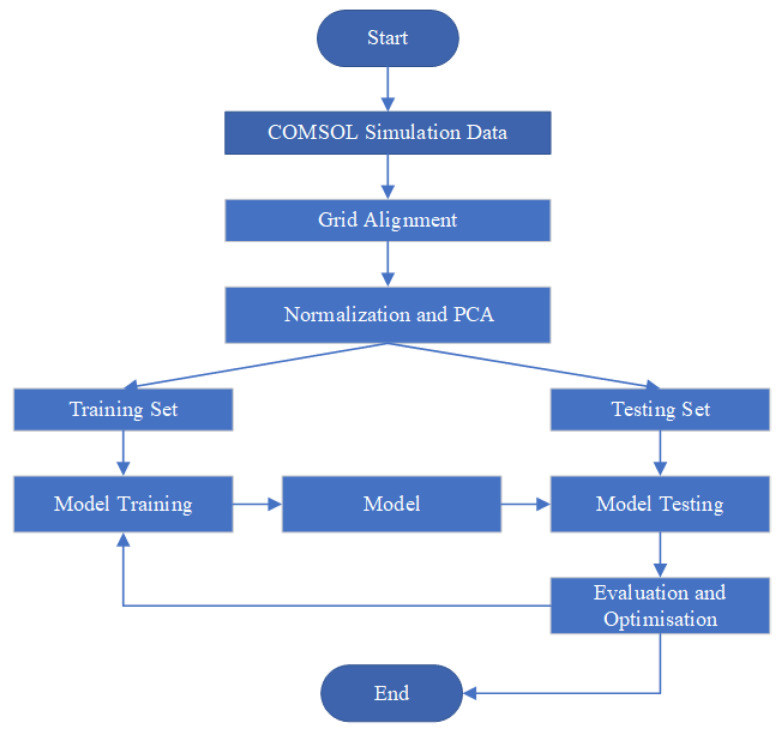
Flowchart of the methodology.

Finally, to demonstrate the advantages of the proposed model, a comparative analysis was made between different models. The overview of the proposed methodology is illustrated in Figure 1.

Our contribution lies in introducing a prediction framework based on machine learning. We evaluated multiple algorithms to determine the most effective magnetic field prediction model for transformers under excitation inrush conditions. This model can predict the magnetic field distribution on a specific set of input features in just 0.41 s.

## 2. Magnetic Field Prediction Model Based on Machine Learning

### 2.1. Random Forest

The Random Forest (RF) algorithm, based on decision trees, typically consists of multiple decision trees and is an ensemble learning method based on Bagging [12]. Decision trees, which are tree-like structures based on information entropy, are able to learn the mapping relationship between features and labels from the data. One of the main advantages of the RF algorithm is its ability to handle large amounts of magnetic data with strong generalization capabilities. Furthermore, RF can perform importance ranking on input features, thereby enhancing the understanding of data variation patterns.

### 2.2. PCA

The PCA is used to reduce the redundancy of data and simplify the magnetic field prediction model. It is difficult to train DNN models by using the high-dimensional output of the original magnetic field. To overcome the limitations of computational resources in practical applications, we have simplified the DNN output by using the PCA method. Through PCA, the original high-dimensional magnetic field simulation data can be effectively represented by the eigenvalues associated with the eigenvectors within a reduced-dimensional K-space. This approach facilitates the concise depiction of the data while maintaining essential information.

The steps of PCA are as follows:

(1) De-meaning the original data matrix.
(2) Computing the covariance matrix of the de-meaned data.
(3) Solving for the eigenvalues and eigenvectors of the covariance matrix.
(4) Sorting the eigenvectors in descending order of eigenvalues.
(5) Selecting the top K eigenvalues and their corresponding eigenvectors based on practical requirements.
(6) Constructing a K-dimensional low-dimensional space and mapping the original data into the low-dimensional space.

### 2.3. XGBoost

The eXtreme Gradient Boosting (XGBoost) is a boosting tree model that integrates many tree models to build a strong classifier, making it superior to traditional Gradient Boosting Decision Trees (GBDT) [13]. The model averts overfitting by incorporating regularization into the objective function. Additionally, in light of the sparsity of the training data, XGBoost can assign a default direction for branches with missing values, which enhances the algorithm’s efficiency. XGBoost also uses the column sampling technique from RF, thereby reducing the computational cost of the model.

### 2.4. DNN

DNN exhibits outstanding feature learning capabilities, extracting features from massive datasets and effectively capturing rich intrinsic information within the data.

This paper employs DNN as a regression tool for generating simulated magnetic field data, mapping magnetic field data to DNN output data. The input is the excitation state, and the output is the dimensionally reduced magnetic simulation data by using PCA.

The training data includes M magnetic field data, each containing K grid points, forming an M×K matrix. After PCA processing, these K dimensions are reduced to P dimensions for training. The prediction results of the DNN are represented by an M×P matrix. The mean, eigenvalues, and eigenvectors of the training data are preserved to perform an inverse PCA transformation on the predicted data, thereby obtaining the predicted magnetic field.

The input is a vector $\left(x_{1},x_{2},...,x_{n}\right)$ and the output is also a vector $\left(y_{1},y_{2},...,y_{m}\right)$; thus, DNN can be considered as a mapping function from the input to the output. This mapping relationship can be discovered by training the neural network to find the correspondence between the input and output, as shown in Equation (1):

(1) $$y=f\left(x,\theta \right)$$

in which θ represents the parameters of DNN, *x* denotes the input vector, and *y* denotes the output vector.

To address the vanishing gradients problem, DNN employs the Rectified Linear Unit (ReLU) function as the activation function for the network layers, thus forming the fundamental framework of DNN. The output of each neuron in layer is computed through the activation function. By modifying the function in the hidden layers in Equation (1), the calculation of the output can be represented as Equation (2):

(2) $$z_{n}=\sigma \left(\left[w_{1}w_{2}...w_{n}\right]{\left[x_{1}x_{2}...x_{n}\right]}^{T}+b_{n}\right)$$

where σ denotes the activation function, $w_{n}$ are the weights connecting the $i^{th}$ neuron from previous layer to the current neuron, $x_{n}$ are the outputs of the $i^{th}$ neuron from the previous layer, $z_{n}$ is the output of the current layer, and $b_{n}$ is the bias added to the current neuron.

The DNN model is trained by using a supervised learning approach. Firstly, an appropriate loss function must be selected for training. Subsequently, the gradient descent method is employed to minimize the model’s loss, thereby achieving the goal of parameter updating.

### 2.5. CNN

Convolutional Neural Network (CNN) is a type of feedforward neural network with a main structure that consists of convolutional layers, pooling layers, and fully connected layers [14]. The basic unit in the convolutional layer is the neuron, which serves as the fundamental unit for learning and training in the neural network. The convolution operation is performed on each kernel within the layer. By computing the convolution with the input vector, the output feature vector of the current convolutional layer is obtained. The convolution computation in CNN is as described by the following equation:

(3) $$y_{l}=\sum_{i=1}^{c^{l-1}}\left(W_{i,c}^{l}*x_{i}^{l-1}+b_{i}^{l}\right)$$

in the equation, $x_{i}^{l-1}$ represents the output of the $i^{th}$ channel in l−1 layer, $c^{l-1}$ denotes the $c^{th}$ channel in l−1 layer, *W* is the weight matrix, and *b* is the bias vector. Similar to DNN models, the parameter values of *W* and *b* are continuously updated and iterated through the process of backpropagation.

### 2.6. Dataset Preparation

**Figure 2:**
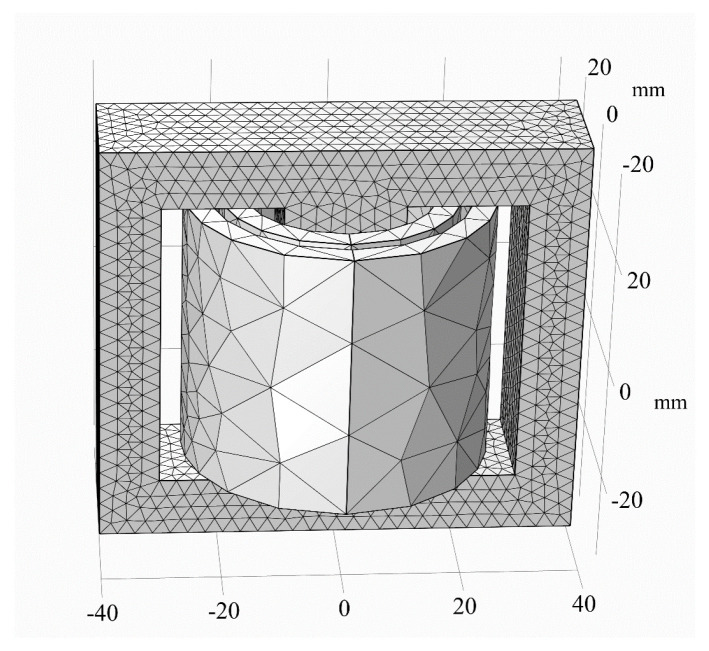
3D physical field mesh diagram of a single-phase transformer.

The excitation inrush state is the condition that transient currents are generated in the windings when the transformer is switched on under no-load conditions, which in turn affects the magnetic field changes in the transformer. For single-phase transformers, whether inrush currents occur and the magnitude of currents are both related to the switching angle. Therefore, the excitation inrush magnetic field simulation data used in this paper are derived from simulations of single-phase transformers with altered switching angles θ, from which the results are obtained. The magnetic core used in our experiments is the B27G120 silicon steel sheet product manufactured by Baosteel. The experiments were conducted under lightly saturated conditions to ensure that the core’s magnetic properties were within the linear region, thereby minimizing non-linear effects that could complicate the analysis. The electrical parameters of a single-phase transformer 3D model in COMSOL 6.0 simulation software are set as listed in Table 1. The physical field mesh model is depicted in Figure 2. The simulation data includes two voltage cycles with time intervals of $5\times 10^{-4}$ s from $5\times 10^{-4}$ s to 0.04 s, with closing angles of 0, $\frac{\pi }{6}$, $\frac{\pi }{3}$, $\frac{\pi }{2}$, $\frac{2\pi }{3}$, $\frac{5\pi }{6}$ and π. Each excitation inrush state includes 80 magnetic field simulation data, totaling 560 data points including seven states, with 10,418 magnetic field mesh points in each data.

As the excitation inrush requires the secondary winding to be in open-circuit state, the current in secondary winding is constantly zero. Therefore, the input features include the induced voltage and current on the primary and secondary windings, as well as the excitation inrush parameter, which denotes the closing angle that controls the magnitude of the excitation inrush, totaling four dimensions. The output data is reduced from the original 10,418 dimensions to P dimensions for training by PCA. The predicted magnetic field of the model are restored by utilizing the eigenvalues and eigenvectors of the training data by inverse PCA transformation.

### 2.7. The Structure of DNN and CNN

**Figure 3:**
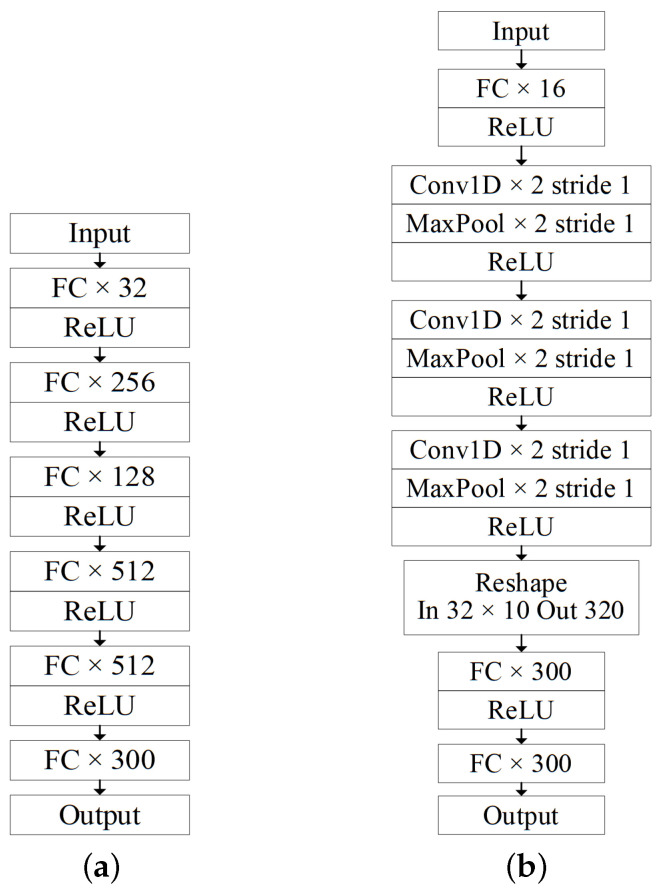
The structure of the excitation inrush transformer magnetic field prediction model. (**a**) DNN. (**b**) CNN.

The Optuna hyperparameter tuning framework is used to select key parameters in the DNN and CNN models, and the network structures of the DNN and CNN models used in this experiment are shown in Figure 3.

The Rectified Linear Unit (ReLU) is used as the activation function for all network layers and the mean absolute error (MAE) was used as the loss function [15], which is defined as follows:

(4) $$MAELoss=\frac{1}{n}\sum_{i=1}^{n}\left|f(x_{i})-y_{i}\right|$$

where $f(x_{i})$ is the predicted value, $y_{i}$ represents the true value, and *n* denotes the number of samples. To assess the performance of models on the testing data,

Mean Absolute Percentage Error (MAPE) is utilized as the evaluation metric. MAPE is the average of the absolute errors between the actual values and the predicted values, divided by the actual values, and it is calculated as shown in Equation (5) [16]:

(5) $$MAPE=\frac{1}{n}\left(\sum_{i=1}^{n}\left|\frac{f(x_{i})-y_{i}}{y_{i}}\right|\right)\times 100\%$$

Additionally, the Mean Squared Error (MSE) and Root Mean Squared Error (RMSE) are also utilized as the evaluation metric. MSE is calculated as shown in Equation (6) [16]. RMSE is calculated as shown in Equation (7) [15]:

(6) $$MSE=\frac{1}{n}\sum_{i=1}^{n}{\left(f(x_{i}\right)-y_{i})}^{2}$$

(7) $$RMSE=\sqrt{\frac{1}{n}\sum_{i=1}^{n}{\left(f(x_{i}\right)-y_{i})}^{2}}$$

Additionally, the R-squared (R^2^), which is often employed as a critical index for regression problems, is shown in Equation (8):

(8) $$R^{2}=1-\frac{\sum_{i=1}^{n}{\left(f(x_{i}\right)-y_{i})}^{2}}{\sum_{i=1}^{n}{\left(\bar{y}-y_{i}\right)}^{2}}$$

where $\bar{y}$ is the average value of true samples.

## 3. Experiments

Before training the predictive model for the magnetic field of inrush currents, it is necessary to analyze the parameter characteristics of the excitation inrush state and to understand their impact on the transformer’s magnetic field. The input feature parameters of this dataset include three dimensions electrical parameters formed by the no-load secondary winding and a parameter representing the transformer’s switching angle, which characterizes the magnitude of the inrush current. The results of feature importance ranking using RF are presented in Table 2

**Figure 4:**
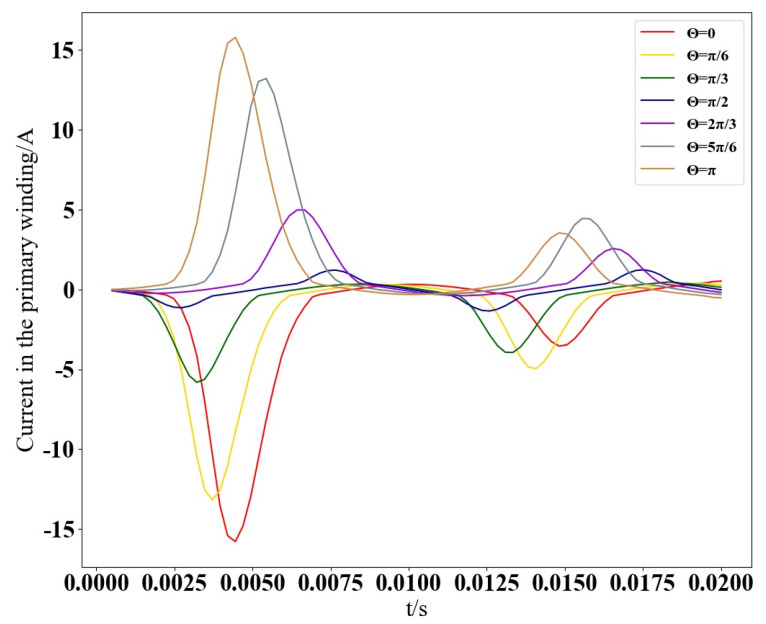
Curves of primary winding current with closing angle.

As indicated in Table 2, the primary winding current has a high importance score of $9.74\times 10^{-1}$, while the closing angle importance is $7.50\times 10^{-3}$, the induced voltage in the primary winding has an importance of $9.40\times 10^{-3}$, and the induced voltage in the secondary winding has an importance of $9.20\times 10^{-3}$. After ranking the feature importance, it was found that the feature with the greatest impact on the magnetic field of single-phase transformers under excitation inrush conditions is the current in the primary winding. To further analyze the effect of the closing angle magnitude on the electrical parameters within a single-phase transformer, the variation curves of the primary winding current at different closing angles are depicted in Figure 4.

Figure 4 demonstrates the impact of different closing angles on the primary winding current. It is observed that as the closing angle transitions from 0 to π, the magnitude and direction of the primary winding current exhibit corresponding changes. Notably, when the closing angle is $\frac{\pi }{2}$, the peak currents in both directions are nearly identical. The inrush currents are maximized at angles of 0 or π, which is reflected in the more obvious peaks in the current waveform. The primary winding current serves as the most direct indicator of the inrush current’s influence. Analysis of the primary winding current’s variations provides fundamental insights into the magnetic field’s behavior under inrush conditions as a function of the closing angle. Consequently, the primary winding current stands out as the paramount input feature for predictive modeling.

**Figure 5:**
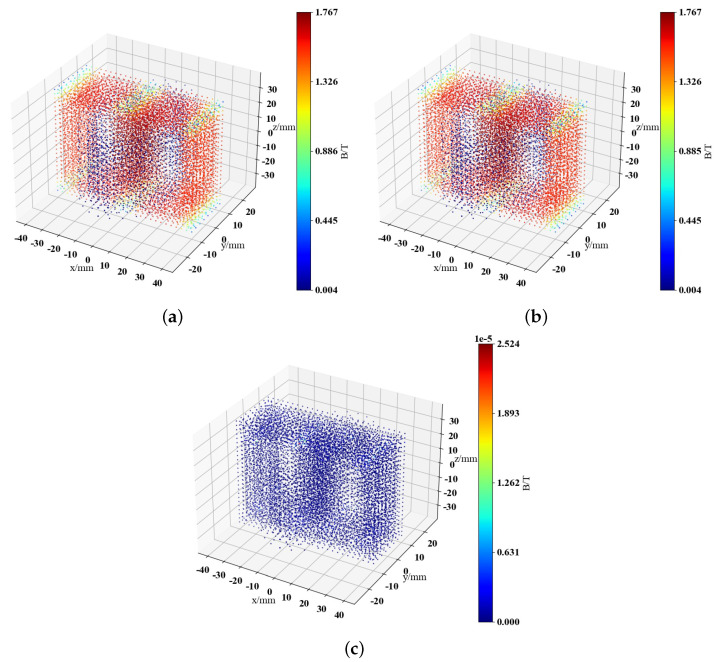
Visual comparison of magnetic field reconstruction results for single-phase transformer with inrush current. (**a**) Training set true values. (**b**) PCA restored results. (**c**) Difference between the two.

The data is dimensionally reduced from the original 10,418 dimensions to 300 dimensions by using PCA. The MAPE between the data restored and the original data is $7.34\times 10^{-3}$%. Figure 5 illustrates the visualization of the magnetic field data at angle $\frac{\pi }{6}$ and time 0.005 s, demonstrating that the difference between magnetic fields obtained after inverse PCA transformation and the original magnetic fields is very small. The maximum discrepancy between the two fields is $1.22\times 10^{-4}$ T, ensuring enough information is preserved.

In this paper, RF, XGBoost, DNN, and CNN models were selected to predict the magnetic field of a single-phase transformer under excitation inrush conditions. After hyperparameter tuning on the RF model, the parameter settings are as follows: n_estimators is 574, max_depth is 432, and max_features is auto. And, the parameter settings on XGBoost model are as follows: n_estimators is 271, max_depth is 7, learning_rate is 0.03, gamma is 0.40, reg_alpha is 0.77, reg_lambda is 0.50, random_state is 95, booster is gbtree, subsample is 0.74, and objective is reg:squarederror.

The Epoch of DNN and CNN are set as 10,000 and Batch Size is 64. The optimizer is Adam, and the learning rate is tuned by using Optuna. The learning rate is decayed every 2500 Epochs during the training process. The four decreasing learning rates are set as $9.28\times 10^{-4}$, $7.37\times 10^{-4}$, $3.24\times 10^{-5}$, $7.07\times 10^{-6}$, respectively.

The input feature dimensionality is 4, the application of convolutional processing would further filter feature information, potentially leading to a degradation in the model’s predictive performance. Therefore, The specific configuration of the CNN model employed for training is as follows: the 1×4 input tensor, following a single fully connected layer, is expanded to a 1×16 tensor. This tensor then undergoes a convolutional module comprising three one-dimensional convolutional layers, each paired with a max-pooling layer, before directly connecting to a fully connected layer. Finally, the output dimensionality is 300. The kernel size for both the convolutional and pooling layers is set to 2, with a stride of 1. ReLU is utilized as the activation function across all network layers. The prediction results from DNN and CNN models underwent inverse PCA transformation and were subsequently compared with the true values. On an experimental setup of the CPU 3.60 GHz, RAM 32 GB, 64-bit Windows 10 operating system, and an RTX 3080 GPU (NVIDIA, Santa Clara, CA, USA), the training time for the DNN model was 61 min and 22 s, while the training time for the CNN model was 63 min and 17 s.

We conducted comparative experiments on multiple models. The results are shown in Table 3.

It can be seen from Table 3 that the excitation inrush single-phase transformer magnetic field prediction model based on DNN has the best performance. The indicators of CNN are very close to those of DNN, indicating that both models have strong applicability to the excitation inrush dataset, while RF and XGBoost perform poorly in all indicators.

**Figure 6:**
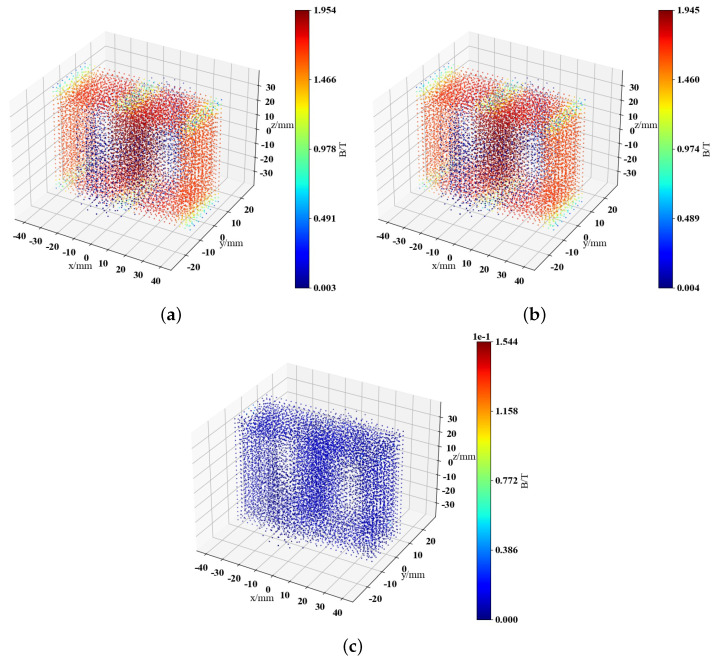
Visualization comparison of magnetic field prediction results of single-phase transformers under excitation inrush conditions. (**a**) Ground truth of the test set. (**b**) DNN prediction results. (**c**) Difference between the two.

The test dataset is set at the closing angle of $\frac{5\pi }{6}$ and a time of 0.0095 s. The magnetic field truth value, DNN model prediction result, and the difference between the two are visualized in Figure 6.

From Figure 6, it can be observed that the maximum value in the error range is $1.54\times 10^{-1}$ T, A comparison reveals that the overall error distribution range is much smaller compared to the original data, and it is concentrated within lower error ranges, achieving high-precision prediction of the magnetic field. In addition, the excitation inrush single-phase transformer magnetic field prediction method proposed in this paper can obtain the distribution of the magnetic field within a specific parameter range much faster than traditional finite element methods, with a prediction time of only 0.41 s by DNN. In contrast, traditional FEA methods take an average of 19.14 s to predict and obtain one set of magnetic field data.

## 4. Conclusions

In this paper, a machine learning-based method was proposed to simulate the magnetic field of a single-phase transformer under excitation inrush, and the performance of different models was analyzed and compared. The effectiveness of the magnetic field prediction method proposed in this paper was validated. Compared to traditional finite element analysis, the method proposed in this paper can efficiently predict the internal magnetic field of transformers, meeting the demand for real-time monitoring of power equipment in smart grid construction. It provides a reference for judging the operating status of equipment and meets the requirements of professional development of power equipment in smart grid applications.

## Figures and Tables

**Table 1:** Single-phase transformer electrical parameters.

Index	Electric Parameter	Value	Index	Electric Parameter	Value
1	Rated voltage	50 V	4	Primary winding turns	300
2	Power supply frequency	50 Hz	5	secondary winding resistance	10 KΩ
3	Primary winding resistance	100 Ω	6	secondary winding turns	300

**Table 2:** Ranking of the importances.

Index	Feature	Importance
1	Primary winding induced voltage	$9.40\times 10^{-3}$
2	Secondary winding induced voltage	$9.20\times 10^{-3}$
3	Primary winding current	$9.74\times 10^{-1}$
4	Closing angle	$7.50\times 10^{-3}$

**Table 3:** Magnetic field prediction results of single-phase transformers under excitation inrush conditions.

Model	MAE (T)	MSE ($T^{2}$)	RMSE (T)	MAPE (%)	$R^{2}$	Param	Time (s)
RF	$2.26\times 10^{-2}$	$7.34\times 10^{-3}$	$8.56\times 10^{-2}$	13.77	0.96		0.01
XGBoost	$2.42\times 10^{-2}$	$6.63\times 10^{-3}$	$8.15\times 10^{-2}$	45.45	0.95		0.07
DNN	$3.75\times 10^{-3}$	$9.25\times 10^{-4}$	$7.62\times 10^{-3}$	4.02	0.99	524 140	0.41
CNN	$5.62\times 10^{-3}$	$1.71\times 10^{-4}$	$2.17\times 10^{-2}$	4.68	0.98	188 048	0.39

## Data Availability

The raw data supporting the conclusions of this article will be made available by the authors on request.
